# A new paradigm of reliable sensing with field-deployed electrochemical sensors integrating data redundancy and source credibility

**DOI:** 10.1038/s41598-022-25920-w

**Published:** 2023-02-22

**Authors:** Ajanta Saha, Sotoudeh Sedaghat, Sarath Gopalakrishnan, Jose Waimin, Aiganym Yermembetova, Nicholas Glassmaker, Charilaos Mousoulis, Ali Shakouri, Alexander Wei, Rahim Rahimi, Muhammad A. Alam

**Affiliations:** 1 School of Electrical and Computer Engineering, Purdue University, West Lafayette, IN 47906 USA; 2 Department of Materials Engineering, Purdue University, West Lafayette, IN 47907 USA; 3 Birck Nanotechnology Center, Purdue University, West Lafayette, IN 47907 USA; 4 Department of Chemistry, Purdue University, West Lafayette, IN 47906 USA

**Keywords:** Engineering, Sensors, Environmental monitoring, Scientific data

## Abstract

For a continuous healthcare or environmental monitoring system, it is essential to reliably sense the analyte concentration reported by electrochemical sensors. However, environmental perturbation, sensor drift, and power-constraint make reliable sensing with wearable and implantable sensors difficult. While most studies focus on improving sensor stability and precision by increasing the system’s complexity and cost, we aim to address this challenge using low-cost sensors. To obtain the desired accuracy from low-cost sensors, we borrow two fundamental concepts from communication theory and computer science. First, inspired by reliable data transmission over a noisy communication channel by incorporating redundancy, we propose to measure the same quantity (i.e., analyte concentration) with multiple sensors. Second, we estimate the true signal by aggregating the output of the sensors based on their credibility, a technique originally developed for “truth discovery” in social sensing applications. We use the Maximum Likelihood Estimation to estimate the true signal and the credibility index of the sensors over time. Using the estimated signal, we develop an on-the-fly drift-correction method to make unreliable sensors reliable by correcting any systematic drifts during operation. Our approach can determine solution pH within 0.09 pH for more than three months by detecting and correcting the gradual drift of pH sensors as a function of gamma-ray irradiation. In the field study, we validate our method by measuring nitrate levels in an agricultural field onsite over 22 days within 0.06 mM of a high-precision laboratory-based sensor. We theoretically demonstrate and numerically validate that our approach can estimate the true signal even when the majority (~ 80%) of the sensors are unreliable. Moreover, by restricting wireless transmission to high-credible sensors, we achieve near-perfect information transfer at a fraction of the energy cost. The high-precision sensing with low-cost sensors at reduced transmission cost will pave the way for pervasive in-field sensing with electrochemical sensors. The approach is general and can improve the accuracy of any field-deployed sensors undergoing drift and degradation during operation.

## Introduction

**Figure 1:**
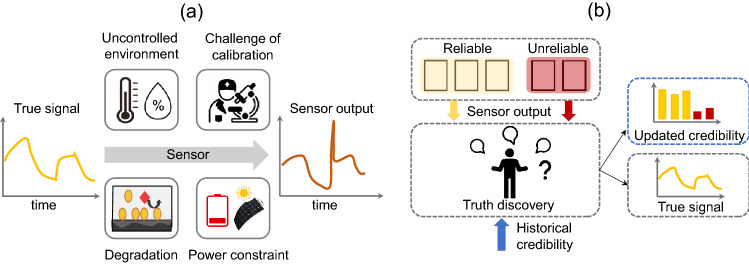
(**a**) Deviation of sensor’s output from the true signal due to several challenges in the field application. (**b**) Schematic of our proposed approach where true signal and sensors’ credibility are estimated using the output and the historical credibility of multiple (reliable and unreliable) sensors.

Reliable sensing with integrated sensors is a prerequisite for continuous monitoring and automatic control systems. Wearable and implantable electrochemical sensors that measure analyte concentrations onsite have potential applications in a variety of systems, e.g., personalized healthcare^1,2^, smart agriculture^3^, wastewater management^4^, chemical, and biological process monitoring^5^, etc. Despite vast opportunities, wearable and implantable electrochemical sensors have not yet attained accuracy levels comparable to those used in the laboratory setting^6,7^. This is because the sensors in field applications face unique challenges absent in laboratory conditions (see Fig. 1a), such as:

- *Uncontrolled environment:* Controlled environment decreases noise and variability, and thus increases the data reliability of the sensors used in the laboratory. In contrast, relatively small sample volume and rapidly varying temperature, humidity, and target concentration increase noise and decrease data reliability of the sensors used in the field^8^.
- *Time-dependent Degradation:* Water ingress, biofouling, radiation exposure, etc., change the physical and chemical properties of the sensor^9,10^. As a result, sensor performance degrades over time when exposed to the natural environment.
- *Challenge of calibration:* Laboratory sensors are frequently calibrated against ground truth signals to correct unwanted drift or bias^11^. Frequent calibration is impossible once the sensor is deployed in the field.
- *Power constraint:* Wired power sources cannot be used for continuous data acquisition and transmission of wearable and implantable sensors. Batteries have a limited power budget and energy harvesters cannot supply power on-demand.

The aforementioned factors adversely affect the accuracy of measurement in the field. To increase the accuracy of measurement, most studies focus on tailoring sensing materials, devices, or systems to make them robust against environmental perturbations and natural degradation^7,12,13^. However, the increased complexity and cost make these sensors unsuited for field applications. In this paper, we offer a complementary sensing paradigm that achieves high-precision measurement by using multiple low-cost sensors that measure the *same* analyte concentration, in a manner analogous to true signal extraction over a noisy communication channel by introducing redundancy in information^14^, see Fig. 1b. Although the use of multiple sensors to achieve high accuracy has been shown in previous works, most of them aggregate sensors’ output based on distance or density of data readings^15,16^. Subsequently, reputation-based data aggregation has been applied to multiple sensors system, where the reputation matrix is defined by the density or distance of data readings of the sensors^17,18^. In these approaches, if a sensor reading deviates from the rest of the group, it is regarded as faulty or an outlier, and excluded during aggregation. Distance or density-based fault detection is effective when faults are rare and significantly different from the normal instances^19,20^. However, it fails when the signal drifts gradually, unreliable readings lie within normal instances, or the majority of the sensors are unreliable. Our aim is to estimate the true signal in presence of both random faults and gradual drift even if the majority of the sensors are unreliable.

To aggregate multiple sensors’ output, we use the concept of truth discovery previously applied in social/crowd/swarm sensing applications^21,22^. In social sensing or crowd sensing problems, often conflicting information is reported by different sources. To discover the truth from this conflicting information, additional information, such as source credibility, reputation, wisdom, or confidence is considered^23–25^. Since the credibility of the sources is not usually known a priori, truth and source credibility are estimated in a coupled way. Truth is estimated by credibility-weighted voting of the sources whereas the credibility of a source is measured by how often or confidently it gives the true information.

In this paper, we adapt this concept of crowd sensing to physical (e.g., electrochemical) sensors. The generalization is nontrivial, because the output of a physical sensor varies both in time and magnitude, while classical social sensing involves binary (true/false) or a few discrete options (multiple choice). We estimate the true signal and credibility index of an individual sensor using the maximum likelihood estimation (MLE) approach^23^. Since wearable and implantable sensors give continuous output in time, we know how a sensor has performed in the past, and how its credibility has changed over time. We augment the historical credibility information to the current estimation to reliably measure the true signal even when the majority of sensors become unreliable (see Fig. 1b). Moreover, using the estimated signals by MLE, we develop an on-the-fly drift correction of unreliable sensors to make them reliable again in field application. We validate our “MLE with drift correction” approach to estimate the true signal and sensor’s credibility with multiple synthetic and real sensor datasets. We theoretically and experimentally validate that historical credibility helps MLE to estimate the true signal when the majority of the sensors are unreliable. Moreover, credibility information allows us to significantly reduce the transmission cost and improve the information yield by suppressing the transmission from unreliable sensors in the wireless sensor network.

## Maximum likelihood estimation

Maximum likelihood estimation (MLE) is a statistical approach to determine target parameters based on observed data. Wang et al. used such an approach for obtaining truth and source credibility in social sensing applications^23^. We adapt this approach for reliable signal estimation from a continuous data stream of electrochemical sensors in field applications. In our case, observed data is the output of multiple sensors, and target parameters are the true signal and the credibility index of each sensor. We divide the continuous output of the sensors into small time windows and MLE assigns high credibility index to a sensor that frequently agrees with the majority at a given time window. Again, it estimates the true signal of that window based on the outputs of the sensors that are assigned high credibility indices. To estimate the true signal and credibility indices in subsequent time windows, we use the average credibility of previous time windows i.e., the historical credibility of the sensors as initialization. Therefore, the true signal and credibility of a time window are estimated based on not only the instantaneous majority but also the historical performance of the sensors. In the Methods section, we describe the MLE framework and integration of historical credibility into MLE, and theoretically show how historical credibility enables true signal estimation even when the majority of the sensors in the network become unreliable.

## Results

In this section, we first validate our MLE based approach to estimate the true signal and credibility of the sensors with synthetic and experimental sensor datasets. Then we show how the estimated signal by MLE can be used for on-the-fly drift correction of unreliable sensors in the field application. Finally, we propose a data transmission technique based on credibility information to reduce transmission energy of wireless sensor networks.

### Dynamic credibility and estimated signal

**Figure 2:**
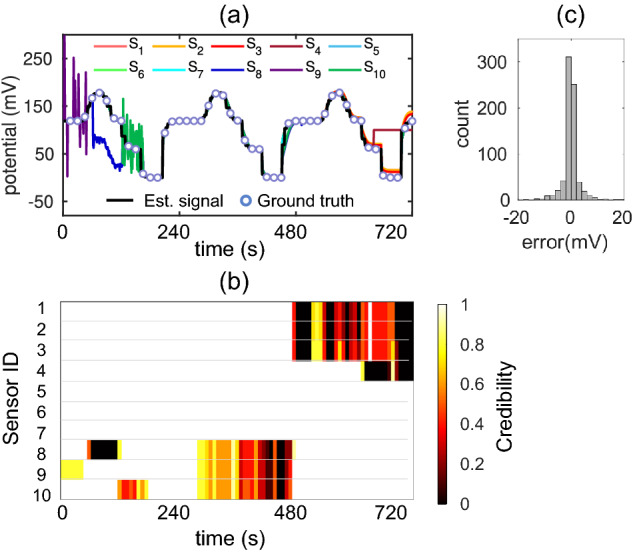
A synthetic dataset. (**a**) The output voltage of the sensors are generated using Nernst equation^26^. Unreliable voltage reading is simulated by changing calibration parameters (e.g., thickness, chemical properties) of the sensors during operation and introducing random fluctuations^28^. Estimated signal by MLE follows the true signal. (**b**) Each sensor’s change of credibility with time. MLE assigns low credibility index to a sensor when a sensor gives unreliable voltage reading and reassign high credibility index when the signal is restored. (**c**) Error distribution between estimated and true signal shows error is close to zero in most instances and RMSE is 5.22 mV.

To validate the MLE approach for the reliable sensing application, we perform experiments on diverse datasets, including synthetic and real sensors. Since our target application is electrochemical sensors, so the synthetic data are generated using analytical equations of Nernst-based ion-selective electrode (ISE). Incidentally, these sensors dominate the electrochemical sensor market^26^. During operation, ISE generates a voltage signal that depends on target ion concentration in the analyte, environmental condition, (e.g., temperature) and calibration parameters (e.g., physical and chemical properties of the sensor)^26^. Figure 2a shows generated voltage signal of 10 ISEs as the analyte concentration is varied in discrete steps. During operation, the calibration parameters of the sensors may change gradually due to water ingress, biofouling, interference with other ions, etc. As a result, these sensors produce unreliable voltage readings^9,27^. To show their effects on voltage signals, we simulate some practical changes in the calibration parameters of unreliable sensors while keeping them constant for reliable sensors. For example, in the time 480 s–740 s, sensors S_1_–S_3_ are sensitive to concentration change but their voltages are shifted by a constant amount due to changes in physical properties. Within the same time-window, S_4_ loses sensitivity abruptly and hence gives a constant voltage reading at the end. Sensors may show delayed response with the concentration change due to the coating of the biofouling layer which we simulate using S_8_–S_10_ during time 300 s–480 s. Other than intrinsic degradation of sensors, extrinsic effects such as power supply or hardware failure, uncontrolled environment, and surrounding perturbation can introduce random fault/fluctuation in the output signal^28^. We consider the effect of random fluctuations in the voltage signal of S_8_–S_10_ in the time period 0 s–160 s. A detailed description of synthetic data generation and fault injection is given in the supplementary information section S1.

MLE takes the voltage signal of reliable and unreliable sensors as inputs and calculates the estimated true signal and each sensor’s change of credibility index with time. Figure 2b validates that the MLE detects all types of unreliability caused by gradual degradation or random fluctuations, and assigns low credibility to the unreliable sensor at that time instance. In this approach, the change in the reliability of the sensors is reflected instantly in the corresponding credibility index. During true signal estimation, unreliable sensors get lower weights, hence the estimated signal follows the reliable sensors’ signal as shown in Fig. 2a. Figure 2c shows the error distribution between the estimated signal and ground truth signal. The error is close to zero most of the time and the RMSE of the estimated signal compared to the true signal is 5.22 mV. For Nernst-based sensors, a 60 mV voltage difference corresponds to about 1 order of magnitude change in analyte concentration. Therefore, the estimated concentration by MLE is within 0.09 orders of magnitude of the true concentration.

### On the fly drift correction

During field operation, a sensor’s signal may drift from the nominal level for various reasons. However, as long as the sensors are nominally sensitive to concentration change of analyte, they are considered reliable. Simple drift correction can provide useful readings from drifted sensors. Such drift or bias correction is done frequently using ground truth signals (as a part of the calibration process) in the laboratory which ensures high precision measurement^11^. Since in the field application, ground truths are not readily available, the estimated signals by MLE can be used as a proxy of the ground truth for drift correction. When most of the sensors are reliable, we have shown with synthetic dataset that the estimated signal by MLE represents the ground truth. Therefore, whenever a sensor drifts, we use the estimated signal as reference to correct the drift of the sensor to make it “reliable” once again. For the drift correction of an unreliable sensor, first, we calculate the error signal between the sensor’s output and the estimated signal by MLE in each time window. Next, we calculate the mean value of the error signal for that time window and shift the output of the drifted sensor by this mean error value, thereby correcting for the drift. The pseudocode of true signal and credibility estimation with drift correction is provided in the supplementary information section S2.

We will now evaluate the performance of MLE algorithm without and with drift correction on three real sensor datasets –

**Figure 3:**
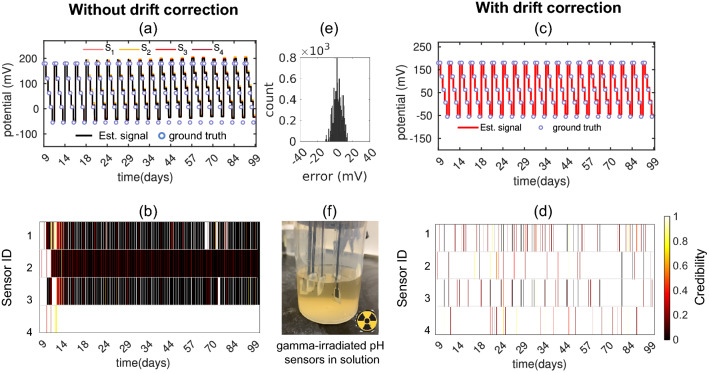
Gradual voltage drift of pH sensors as a function of gamma (γ)-irradiation. (**a**) Recorded voltage of four pH sensors for more than 3 months after being exposed to γ-irradiation (25–45 kGy) at day 0^30^. Due to divergence of individual voltage readings estimated signal by MLE deviates from ground truth at later days and (**b**) MLE assigns low credibility to majority of the sensors. After drift correction, (**c**) estimated signal by MLE follows the ground truth signal more precisely and (**d**) high credibility is assigned to each sensor. (**e**) Error distribution between estimated (after drift correction) and true signal shows error is negligible in most instances with RMSE 5.74 mV. (**f**) γ-irradiated pH sensors stored in buffer solution.

**Figure 4:**
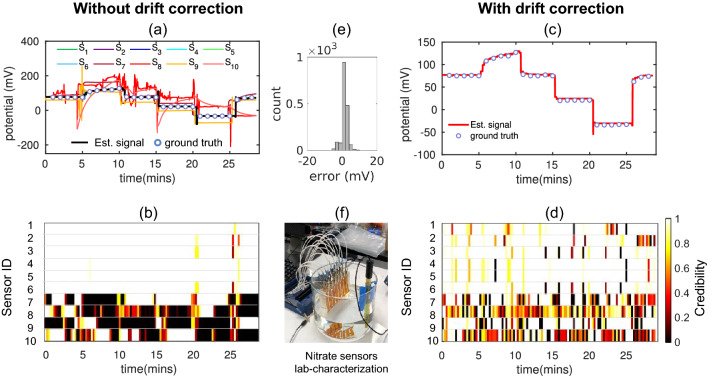
Random faults due to electrical noise and failure of sensors. (**a**) Recorded voltage readings of 10 roll-to-roll printed nitrate sensors in controlled experiment where random faults in S_7_–S_10_ are observed due to manufacturing defects or electrical noise. Estimated signal by MLE follows the true signal. (**b**) MLE assigns low credibility index to S_7_–S_10_ when they fail to follow the true signal. (**c**) Estimated signal by “MLE with drift correction” along with ground truth signal. (**d**) Drift correction corrects the bias of S_7_ and S_9_ thereby they get high credibility index after drift correction. (**e**) Error distribution between estimated (after drift correction) and true signal shows error is close to zero most of the time instances. (**f**) Experimental setup of laboratory characterization of the Nitrate sensors.

**Figure 5:**
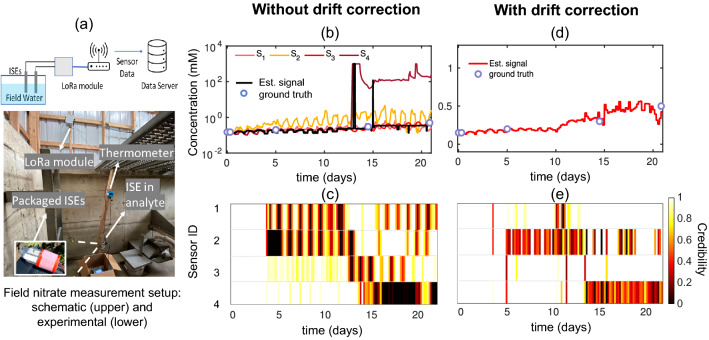
(**a**) Schematic (upper) and experimental (lower) setup of field measurement. (**b**) Nitrate concentration of an agricultural field is measured continuously with four low-cost nitrate ISEs over a period of 22 days. Part of the voltage readings are adapted from Ref ^8,33^. Different faults are observed- slow drift over time (S_1_), increasing sensitivity to temperature with time (S_2_), sudden jump in output (S_4_). If we do not correct drifts, three sensors become unreliable after 12–13 days and MLE fails to estimate correct concentration and credibility. (**c**) MLE assigns high credibility to S_2_ although it is not reliable at later days. Again, S_3_ is assigned low credibility at certain times although it is reliable. (**d**) Drift correction corrects the drift ahead of time, so that drift-corrected estimated signal follows the ground truth more accurately. (**e**) After drift correction, MLE correctly assigns lower credibility to S_2_ and S_4_.

- *Gradual drift due to exposure to gamma-ray irradiation:* One emerging application for electrochemical sensors is monitoring of analytes in bioreactors, which are typically sterilized by gamma (γ)-irradiation^5^. However, when ISEs are exposed to γ-irradiation during in-situ monitoring, their chemical properties are altered^29^, resulting in a change in voltage drift behavior^30^. We use MLE to estimate the analyte concentration accurately even if sensor’s voltage drifts gradually as a function of γ-irradiation. For that, we monitor the response of four γ-irradiated pH sensors stored for more than three months in buffer solution (Fig. 3f). To quantify the gradual signal drift, we periodically measure voltage of the sensors with respect to commercial Ag/AgCl reference electrode in pH5–pH9 (2 min in each solution). The details about pH sensor fabrication and characterization process can be found in Ref^30^. Figure 3a shows systematic divergence of individual voltage readings from the initial calibration curve (i.e., ground truth) with time. We assume that the ground truth voltage of subsequent measurements is the same as the first measurement since we make sure that pH values of solutions are exactly same at each measurement. Therefore, the ground truth should be constant across all measurements if there is no voltage drift of the sensors. Since the MLE algorithm does not use any ground truth supervision to estimate the true signal and credibility of the sensors, it cannot recover the true signal if all the sensors diverge from each other and the ground truth. Therefore, the estimated signal by MLE shows a deviation from the true signal and low credibility is assigned to majority of the sensors (Fig. 3b). Although MLE cannot recover the true signal in this case, our drift correction algorithm corrects the small divergences at each step which leads MLE to the correct estimation afterward.
  Figure 3a shows that after being exposed to gamma-ray irradiation, sensors still respond correctly to the change of pH, only that their voltage level shifts gradually over time, which can be corrected by the drift correction algorithm. As a result, after drift correction, all the sensors contribute toward a reliable estimation, and MLE correctly assigns high credibility to the sensors as shown in Fig. 3d. Figure 3c shows that the estimated signal by MLE more precisely follows the ground-truth signal after drift correction with negligible error at the most time instances (see error distribution in Fig. 3e). Without drift correction, RMSE of the estimated signal was 14.22 mV; the RMSE is reduced to 5.74 mV after drift correction. It implies that, even without any recalibration, “MLE with drift correction” can estimate the pH of the solution within 0.09 pH for more than three months after these Nernst-based pH sensors are exposed to γ-irradiation.
- *Random faults in the controlled experiments:* For continuous monitoring of nitrate concentration in the agricultural field, we developed implantable, low-cost Nernst-based nitrate ISEs by using a roll-to-roll process. The nitrate sensor fabrication details can be found in Ref^26,31^. Before field deployment, the sensors are characterized by controlled laboratory experiments where the voltage signal of each sensor is recorded with respect to the commercial Ag/AgCl reference electrode by varying the nitrate concentration of the solution in discrete steps (Fig. 4a). The response of a commercial nitrate sensor is recorded as a ground-truth signal. Some low-cost sensors (e.g., S_7_–S_10_) failed to follow the true signal due to sensor failure or external electrical noise during the experiment. We denote the faults as “random faults” and detect them using MLE. Figure 4b shows MLE correctly assigns low credibility to the faulty sensors S_7_-S_10_. As a result, the estimated signal closely follows the ground-truth signal (Fig. 4a) and RMSE of the estimated signal compared to the ground-truth signal is 5.26 mV.
  Since S_8_ and S_10_ fail randomly as shown in Fig. 4a, MLE marks them as unreliable even after drift correction, which is shown in dynamic credibility index of Fig. 4d. On the other hand, the constant shifts of S_7_ and S_9_ can be corrected in this method, and they are assigned high credibility after drift correction. Finally, Fig. 4c shows that the estimated signal after drift correction closely follows the ground truth signal with RMSE 3.51 mV. In other words, “MLE with drift correction” can estimate the concentration within 0.06 orders of magnitude of true concentration.
- *In-situ nitrate concentration measurement in the agricultural field:* Finally, we apply the MLE approach for high accuracy measurement of nitrate concentration in an agricultural field with multiple low-cost nitrate sensors. The experimental setup of field deployment is described in the supplementary information section S3 and shown in Fig. 5a. In the field, sensors undergo additional challenges, e.g., interference of secondary ions, biofouling, natural variation of temperature between day and night, etc., which makes it challenging to achieve reliable sensing in field application. Figure 5b shows calculated concentrations from voltage readings of four nitrate sensors in the field solution. Ground truth points are measured by a laboratory-based high-precision nitrate sensor^32^. Different types of faults are observed, for example, S_1_ slowly drifts with time, S_2_ becomes more sensitive to surrounding temperature after a few days, and the output level of S_4_ shifts after 13 days. Figure 5b shows the estimated signal by MLE follows the trend of ground truth upto 12–13 days. After that, MLE gives random values at some time instances. We also see from Fig. 5c that although S_2_ becomes unreliable at later days, MLE fails to assign low credibility to S_2_. MLE fails to estimate the true signal and the sensor credibility in this period because after 12–13 days only S_3_ remains reliable, the other three sensors (i.e., S_1_, S_2_, and S_4_) become partially or fully unreliable. Without ground truth supervision, MLE cannot recover the true signal if a minimum number of sensors are not reliable. However, through drift correction, we correct the drift of the unreliable sensors ahead of time using the MLE signal, so that we can later ensure the minimum number of reliable sensors required for MLE to function appropriately.
  Drift correction helps to get much more interpretable results with field-deployed nitrate sensors (see Fig. 5d,e). Drift corrections keep more sensors nominally reliable over time. As a result, MLE can correctly detect S_2_ and S_4_ as unreliable sensors and accordingly assign low credibility to these sensors. Estimated signal also closely follows the trend of ground truth even after 12–13 days. We obtain RMSE between estimated signal and interpolated ground truth-signal to be 0.06 mM over the period of 22 days. We know of no other report in the literature that continues to provide a reliable signal in the field after such a long-term operation.

### Data transmission based on credibility

Wearable and implantable sensors are usually powered by battery or by natural energy harvesters. Signal transmission is the primary source of the energy cost of a sensor (local signal processing costs are orders of magnitude lower^34^). To reduce the power consumption of data acquisition and transmission, different strategies are taken. One common strategy is to operate the sensors at a low duty cycle, i.e., sensors send data in long time intervals^35^. However, sensor may miss some important events during the long interval. To overcome this shortcoming, event-based data transmission is proposed in previous works^36,37^. In classical event-based transmission, when a data point differs from the previous event by more than a predefined threshold, the data point is detected as an event and transmitted^36^. However, in this approach, any random faults or gradual shift of unreliable sensors can be detected as an “event” and the unreliable data point can be sent to the receiver. This spurious transmission not only reduces information yield but also increases the transmission cost. We propose to integrate the credibility information provided by MLE with the event-based data transmission technique to eliminate unnecessary data transmission from unreliable sensors.

**Figure 6:**
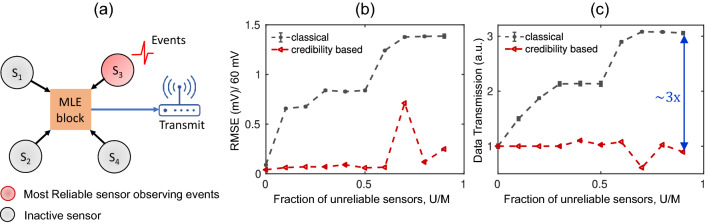
(**a**) Schematic of credibility-based data transmission framework. Comparison of (**b**) RMSE and (**c**) data transmission (relative to data transmission when all sensors are reliable), in classical ^36^ and credibility based data transmission approach. Fraction of unreliable sensors is varied in real sensor dataset keeping total sensor fixed at 10. When all sensors regardless of credibility are allowed to send data, RMSE and data transmission increase significantly even with a few unreliable sensors in the network. Whereas these quantities are reduced significantly by sending data based on the algorithm proposed in this paper.

The proposed data transmission approach has two parts: updating of credibility index and detecting of signal events. The schematic of data transmission approach is shown in Fig. 6a. For the update of the credibility index, we collect the signals of all sensors for a time window and estimate the credibility of the sensors by MLE. Based on this credibility, we select the most reliable sensor which will monitor the events until the next credibility update. Rest of the sensors remain inactive i.e., do not observe events. For detecting signal events, an event detection threshold is defined as suggested in^36^. The most reliable sensor detects an event if the data point differs from the last transmitted data point by more than the threshold value. When an event is detected, all the sensors start collecting data for a time window and send data to the MLE block where we run MLE to update the sensors’ credibility, estimate true signal of that time window, and correct drift of unreliable sensors using the MLE signal. If the sensor that has detected event is most reliable sensor based on updated credibility, then estimated signal by MLE is transmitted to the receiver. If the sensor is no longer the most reliable sensor of the group, then no data is transmitted. In this way, if the most reliable sensor becomes unreliable during the period of event detection and detects a false event, then no data will be transmitted. Based on updated credibility, the most reliable sensor will take over event detection and others will become inactive. Pseudocode of the data transmission algorithm is provided in the supplementary information section S4. We obtain the transmitted data from sensors’ signal using the algorithm and recover the signal by interpolating the transmitted data. Information yield is defined by the RMSE between true signal and recovered signal, where reduced RMSE implies enhanced information yield. We compare the results with classical event-based transmission, where all sensors observe events and whenever a sensor detects an event, it can send data regardless of its credibility^36^.

The comparison between classical event-based data transmission approach and our proposed approach in terms of number of data transmitted and RMSE is summarized in Table 1 for three real sensor datasets (in Figs. 3, 4, 5). The event detection threshold used for each dataset is reported in the supplementary information Table S1. For gradual drift of γ-irradiated sensors, the use of credibility information does not reduce the data transmission significantly because here the unreliable sensors are slowly drifting with time, so they are not detecting and sending false events (Fig. 3a). However, the RMSE is reduced by ~ 2 × when the estimated signal by MLE is sent. For the second dataset (Fig. 4a), unreliable sensors failed randomly which are detected as events in classical event-based technique, and so data transmission increases significantly if we allow them to send the false events. Credibility based approach reduces the data transmission and improves the information yield (reduces RMSE) significantly by sending only the reliable data. For the field data, we achieve ~ 2 × reduction in data transmission and a significant reduction in RMSE using our approach.

**Table 1:** Comparison between classical^36^ and credibility-based data transmission approach in terms of no data sent and RMSE (between recovered and true signal) for three real sensor datasets**.**

Real sensor datasets	No of data sent		RMSE	
	Classical	Credibility based	Classical	Credibility based
Gradual drift	5023	4105	17.19 mV	10.24 mV
Random faults	2055	790	49.54 mV	3.87 mV
Field	964	584	1504 mM	0.067 mM

Finally, we evaluate the effect of the fraction of unreliable sensors on RMSE and the data transmission by increasing the number of unreliable sensors while keeping the total number of sensors fixed in the network. We use the measured voltages from our roll-to-roll manufactured, low-cost nitrate sensors in the laboratory settings for the analysis. The response of a commercial nitrate sensor is recorded as the ground truth signal. The sensors that follow the signal of the commercial nitrate sensor are regarded as reliable sensors, and others that show random faults or gradual drift are regarded as unreliable ones. The datasets used for the analysis is given in the Supplementary information Fig. S1. Figure 6b and c show the comparison of RMSE and the data transmission between our approach vs. the classical event-based approach, while fraction of unreliable sensors is increased keeping total number of sensors fixed at 10. Here, the mean of RMSE and data transmission (along with corresponding standard deviations) are reported for 5 different runs. As expected, when all the sensors are credible, the RMSE and the data transmission rates are the same whether we use credibility information or not. In the classical approach, since all sensors are allowed to transmit data regardless of their credibility indices, RMSE and data transmission increase rapidly even with relatively few unreliable sensors in the network. Since 60 mV voltage difference corresponds to 1 order magnitude difference in concentration, the estimated concentration is off by ~ 0.7 orders of magnitude with only one unreliable sensor. Data transmission and RMSE increase because unreliable sensors detect false events and send incorrect data. When we allow only the most credible sensor to monitor an event and send reliable data, we can keep the RMSE small, and thereby estimate the concentration accurately even when 90% of sensors become unreliable. We can simultaneously reduce the data transmission by two-thirds compared to classical event detection method (which by itself reduces data transmission by an order magnitude compared to continuous transmission of the data). Only at fraction of unreliable sensor = 0.7, we observe comparatively large RMSE because the algorithm fails to detect some true events.

## Discussion

### Integration of historical credibility leads MLE to more informative answer

**Figure 7:**
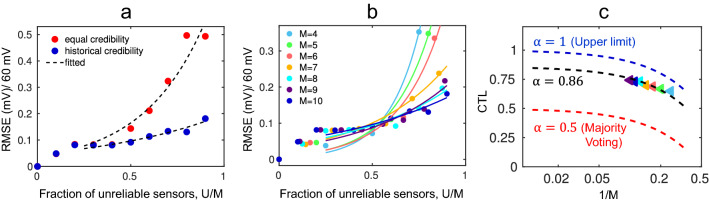
(**a**) Effect of historical credibility information in initialization at each time window- RMSE between estimated signal by MLE and true signal denotes when historical credibility information is used for initialization of MLE, RMSE decreases significantly for fraction of unreliable sensor greater than 0.5 (number of unreliable sensor, U and total sensor, M = 10). (**b**) RMSE using historical credibility vs. fraction of unreliable sensors for total number of sensors, M = 4–10. (**c**) Credibility threshold limit (CTL) is obtained by fitting RMSE datapoints with Eq. (1). We can obtain CTL close to the upper limit using historical credibility with MLE.

For time-invariant ground-truth data related to typical social sensing applications, MLE estimates the credibility of a source based on the whole dataset and uses the credibility to estimate the truth correctly^23^. However, for continuous time-series data discussed in this paper, we need to estimate the signal window-wise using current or small-time interval data. In that case, the performance within a single time window is not sufficient to determine the sensor’s credibility, especially if the time window is relatively small. As a result, if all sensors are assumed to be equally reliable during initialization at each time window, the MLE technique is reduced to simple majority voting. Majority voting is useful when most of the sensors are reliable. However, it fails to estimate the true signal correctly when majority are unreliable. We use the historical credibility information for initialization of current time window to bias the estimation towards the sensors that showed higher credibility in the past. If reliable ones are few in number, this leads to more accurate estimation compared to the MLE with the assumption of equal credibility of all sensors. We validate the effect of historical credibility with the nitrate sensor dataset that showed gradual drift and random faults during laboratory measurement (shown in the supplementary information Fig. S1). Figure 7a shows the RMSE of estimated signal compared to ground truth signal measured by commercial nitrate sensor while fraction of unreliable sensors is increased, but keeping total number of sensors fixed at 10. When fraction of unreliable sensors is less than 0.5 i.e., reliable sensors are majority, the RMSE is comparable whether we assume that all sensors are equally reliable (red circles) or use the historical credibility information (blue circles). However, when majority is unreliable, RMSE increases exponentially when there is no memory of historical performance. The use of the historical credibility reduces the RMSE significantly when fraction of unreliable sensors is greater than 0.5. Figure 7a shows that even when 90% of sensors are unreliable, the analyte concentration can be estimated within 0.2 orders of magnitude by using historical credibility information.

### Integration of historical credibility increases credibility threshold limit of MLE

Since MLE algorithm does not use any ground-truth supervision for estimating the true signal and the credibility of the sensors, it cannot recover the true signal if all the sensors simultaneously become unreliable. Few sensors must be reliable to recover the true signal. The maximum fraction of unreliable sensors with which MLE can still correctly detect the true signal and determine the sensor’s credibility defines the credibility threshold limit (CTL) of MLE. When the fraction of unreliable sensor is greater than CTL, RMSE increases significantly. The CTL of the simple majority voting algorithm is 0.5, whereas in the Methods section, we theoretically show by integrating the historical credibility, MLE can achieve CTL greater than 0.5. To numerically calculate the CTL of the group of sensors used in Fig. 7a, we fit the RMSE data points obtained by using historical credibility to MLE approach (blue circles) with Eq. (1),

1 $${\text{RMSE}} = {\text{C}} \times \exp \left( {\frac{{{\text{u}} - {\text{CTL}}}}{{{\text{u}}_{0} }}} \right)$$

where ${\text{u}} = {\text{U}}/{\text{M}}$ is the fraction of unreliable sensors in the sensor group with total number of sensors ${\text{M }}$ and number of unreliable sensors ${\text{U}}$. The values of ${\text{C}}$, ${\text{u}}_{0}$ and CTL are obtained from the fitted line. According to Eq. (1), the RMSE increases exponentially with ${\text{u}} > {\text{CTL}}$. Following this, we calculate CTLs by fitting RMSE versus ${\text{u}}$ datapoints by varying the total number of sensors in the group. Figure 7b shows the fitted lines for total number of sensors, M = 4–10. Figure 7c plots the CTLs as a function of $1/{\text{M}}$ (triangles). CTL is plotted as a function of $1/{\text{M}}$ to show the asymptotic value of CTL when ${\text{M}}$ is very large. CTL can be expressed as a function of total number of sensors, ${\text{M}}$ as follows,

2 $${\text{CTL}} = \frac{{{\alpha \rm M} - 1}}{{\text{M}}}$$

where ${\upalpha }$ is the asymptotic value of CTL (${\text{M}} \to \infty$) which varies between 0.5 and 1. For majority voting, ${\upalpha } = 0.5$ which says that the maximum fraction of unreliable sensors is 0.5 for reliable signal extraction. If only one reliable sensor can extract the reliable signal, then ${\upalpha } = 1$ which is theoretical upper limit of ${\upalpha }$. By fitting the numerical CTL points with Eq. (2), we obtain ${\upalpha } = 0.86$ (see Fig. 7c). Therefore, using historical credibility, we obtain ${\upalpha }$ close to the upper limit. In other words, we can estimate the true signal even when more than 80% of sensors become unreliable. The value of ${\upalpha }$ depends on the signal quality of the sensors. If majority of the sensors are unreliable from the beginning or become unreliable at the same time, then historical credibility cannot help the MLE algorithm to estimate the correct signal. Again, if malicious attackers modify the highly reliable sensor reading in a planned way, then our approach will not be able to detect it. If the sensors become unreliable naturally at different times, as is the case for most field-deployed sensors, then the proposed MLE approach will give high credibility to the reliable majorities at the beginning. The high historical credibility of reliable sensors will help MLE to follow them even if majority becomes unreliable at later times.

## Conclusion

We have developed a novel approach to obtain reliable data from low-cost electrochemical sensors in the field application using data redundancy and sensors’ credibility based on MLE. With multiple synthetic and real sensor datasets, we have demonstrated that we can detect the unreliable sensors and estimate the true signal even when some of the sensors become unreliable either due to gradual degradation or random faults. We have developed an on-the-fly drift correction method to correct the drift or bias of the sensors during operation. Once corrected, these sensors become reliable once again. Using the γ-irradiated  pH sensor dataset, we show that we can measure the pH of the solution within 0.09 pH for more than three months without any external recalibration. The approach has also been applied for measuring nitrate concentration of an agricultural field onsite with multiple low-cost nitrate sensors within 0.06 mM of high-precision laboratory-based sensors over a period of 22 days. We propose an algorithm to integrate credibility information into the classical event-based data transmission approach. Using real sensor datasets, we have shown that the algorithm can reduce data transmission and concentration estimation error significantly by suppressing unnecessary data transmission from unreliable sensors. Finally, we theoretically show that MLE can estimate the true signal and credibility of the sensors correctly by using the historical credibility of the sensors even if more than half of the sensors are unreliable. Indeed, we achieve CTL greater than 0.8 for a real sensor dataset consisting of unreliable sensors with random faults or gradual drift.

Our MLE-based approach is scalable and results are explainable. The approach does not require expensive sensors and large data transmission, and yet shows robustness when most sensors have random faults and gradual drift; all these make the approach suitable for a variety of field applications. However, as we do not use any ground truth and physical supervision, but rather rely on historical credibility, this approach will not be able to estimate the true signal if all the reliable sensors fail at the same time or are modified in a planned way. In this situation, periodic supervision from a high-precision sensor or another secondary variable can be helpful to detect and correct such faults. We will explore this topic in our future research. Although this paper focused on electrochemical sensors, this is a general algorithm that can be used to estimate data reliability of any data source for applications in analytical modeling, statistical inference, and prediction from machine learning algorithms.

## Methods

### Inputs and outputs

MLE takes reliable and unreliable signals from the sensors as inputs and calculates the estimated signal and the credibility of the sensors as outputs. Since a sensor’s signal is a continuous streaming data, we split the input signal in finite sized time windows and then use a sliding window to keep the memory of previous performance. Let us assume that ${\text{SC }} \in { }{\mathbb{R}}^{{{\text{M}} \times {\text{N}}}}$ is the input matrix at a time window, where ${\text{M}}$ is the number of sensors and ${\text{N}}$ is the window size. Since sensors’ readings can take continuous value, we divide the entire range of sensors’ readings of a time window into ${\text{K}}$ discrete levels and quantize each reading to the nearest level. The output of MLE for a time window is estimated signal at each time index, ${\text{O }} \in { }{\mathbb{R}}^{{\text{N}}}$ and credibility indices of the sensors, ${\text{t}} \in { }{\mathbb{R}}^{{\text{M}}}$. We solve the problem with Expectation Maximization algorithm as suggested by Wang et al.^23^. Pseudocode of the true signal and credibility estimation is provided in supplementary information section S2 and window size, overlap size, and quantization levels used in the analysis of synthetic and real sensor datasets (in Figs. 2, 3, 4, 5) are reported in the supplementary information Table S2. We also show the impact of varying window size, ${\text{N}}$ and quantization levels, ${\text{K}}$ on the quality of estimated signal from MLE in the supplementary information Fig. S2 where small ${\text{N}}$ and ${\text{K}}$ are desired to reduce computational complexity.

### True signal and credibility estimation

Keeping in consistent with the notations of^23^, we assume that ${\text{S}}_{{\text{i}}}$ represents ith sensor and ${\text{C}}_{{\text{j}}}$ represents jth time index in the input matrix, ${\text{SC}}$. ${\text{S}}_{{\text{i}}} {\text{C}}_{{\text{j}}} = {\text{k}}$ denotes ${\text{S}}_{{\text{i}}}$ measures ${\text{C}}_{{\text{j}}}$’s value to be ${\text{k}}$, where ${\text{k}} = 1,2, \ldots .,{\text{K}}$. Similarly, ${\text{S}}_{{\text{i}}} {\text{C}}_{{\text{j}}} = {\overline{\text{k}}}$ means it measures the value other than ${\text{k}}$. ${\text{s}}_{{\text{i}}}^{{\text{k}}}$ is the probability that ${\text{S}}_{{\text{i}}}$ measures a value to be ${\text{k}}$ (i.e., ${\text{s}}_{{\text{i}}}^{{\text{k}}} = {\text{P}}\left( {{\text{S}}_{{\text{i}}} {\text{C}}_{{\text{j}}} = {\text{k}}} \right)$) and ${\text{s}}_{{\text{i}}}^{{{\overline{\text{k}}}}}$ is the probability to measure other than ${\text{k}}$ (i.e., ${\text{s}}_{{\text{i}}}^{{{\overline{\text{k}}}}} = {\text{P}}\left( {{\text{S}}_{{\text{i}}} {\text{C}}_{{\text{j}}} \ne {\text{k}}} \right)$). Moreover, ${\text{t}}_{{\text{i}}}^{{\text{k}}}$ is credibility of sensor ${\text{S}}_{{\text{i}}}$ which is defined as the probability that ${\text{C}}_{{\text{j}}}$’s value is ${\text{k}}$ given that ${\text{S}}_{{\text{i}}} {\text{C}}_{{\text{j}}} = {\text{k}}$ (i.e., ${\text{t}}_{{\text{i}}}^{{\text{k}}} = {\text{P}}\left( {{\text{C}}_{{\text{j}}} = {\text{k|S}}_{{\text{i}}} {\text{C}}_{{\text{j}}} = {\text{k}}} \right)$).

Furthermore, ${\text{a}}_{{{\text{k}},{\text{i}}}}^{{\text{T}}}$ and ${\text{a}}_{{{\text{k}},{\text{i}}}}^{{\text{F}}}$ are defined as the probability that ${\text{S}}_{{\text{i}}}$ measures a value to be ${\text{k}}$ and other than ${\text{k}}$ respectively when it is indeed ${\text{k}}$, i.e.,

3 $$\begin{aligned} {\text{a}}_{{{\text{k}},{\text{i}}}}^{{\text{T}}} & = {\text{P}}\left( {{\text{S}}_{{\text{i}}} {\text{C}}_{{\text{j}}} = {\text{k|C}}_{{\text{j}}} = {\text{k}}} \right) \\ {\text{a}}_{{{\text{k}},{\text{i}}}}^{{\text{F}}} & = \mathop \sum \limits_{{\overline{\text{k}} \ne {\text{k}}}} {\text{P}}\left( {{\text{S}}_{{\text{i}}} {\text{C}}_{{\text{j}}} = \overline{\text{k}}{\text{|C}}_{{\text{j}}} = {\text{k}}} \right) \\ \end{aligned}$$

Since sensors will always measure one or another value, ${\text{a}}_{{{\text{k}},{\text{i}}}}^{{\text{T}}} + {\text{a}}_{{{\text{k}},{\text{i}}}}^{{\text{F}}} = 1$. Finally, ${\text{d}}_{{\text{k}}}$ is the prior probability that signal value at any time is k. Applying Bayes theorem to Eq. (3),

4 $${\text{a}}_{{{\text{k}},{\text{i}}}}^{{\text{T}}} = \frac{{{\text{t}}_{{\text{i}}}^{{\text{k}}} \times {\text{s}}_{{\text{i}}}^{{\text{k}}} }}{{{\text{d}}_{{\text{k}}} }}$$

Therefore, ${\text{a}}_{{{\text{k}},{\text{i}}}}^{{\text{T}}}$ is proportional to sensors credibility ${\text{t}}_{{\text{i}}}^{{\text{k}}}$.The estimation parameter of MLE is ${\uptheta }_{{\text{k}}} = \left( {{\text{a}}_{{{\text{k}},{\text{i}}}}^{{\text{T}}} { },{\text{ d}}_{{\text{k}}} } \right)$, for ${\text{i}} = 1,2, \ldots ,{\text{M}}$ and ${\text{k}} = 1,2, \ldots .,{\text{K}}$. A latent variable Z is defined for the estimated true value at each time index. For a given input matrix $X = {\text{SC}}$, estimation parameter, ${\uptheta }$ and latent variable, ${\text{Z}}$, the likelihood function is given by,

5 $${\text{L}}\left( {{\uptheta }:{\text{X}},{\text{Z}}} \right) = {\text{P}}\left( {{\text{X}},{\text{Z}}|\uptheta } \right) = \mathop \prod \limits_{{{\text{j}} = 1}}^{{\text{N}}} \left\{ {\mathop \sum \limits_{{{\text{k}} = 1}}^{{\text{K}}} \left[ {\mathop \prod \limits_{{{\text{i}} = 1}}^{{\text{M}}} ({\text{a}}_{{{\text{k}},{\text{i}}}}^{{\text{T}}} )^{{{\text{S}}_{{\text{i}}} {\text{C}}_{{\text{j}}}^{{\text{k}}} }} \times ({\text{a}}_{{{\text{k}},{\text{i}}}}^{{\text{F}}} )^{{{\text{S}}_{{\text{i}}} {\text{C}}_{{\text{j}}}^{{{\overline{\text{k}}}}} }} \times {\text{d}}_{{\text{k}}} \times {\text{z}}_{{\text{j}}}^{{\text{k}}} } \right]} \right\}$$

where ${\text{S}}_{{\text{i}}} {\text{C}}_{{\text{j}}}^{{\text{k}}} = 1$ when ${\text{S}}_{{\text{i}}}$ measures ${\text{C}}_{{\text{j}}}$’s value to be ${\text{k}}$ (i.e., ${\text{S}}_{{\text{i}}} {\text{C}}_{{\text{j}}} { } = {\text{ k}}$) and 0 otherwise. Similarly, ${\text{S}}_{{\text{i}}} {\text{C}}_{{\text{j}}}^{{{\overline{\text{k}}}}} = 1$ when ${\text{S}}_{{\text{i}}} {\text{C}}_{{\text{j}}} { } \ne {\text{k}}$ and 0 otherwise. ${\text{z}}_{{\text{j}}}^{{\text{k}}} = 1$ when ${\text{C}}_{{\text{j}}}$’s value is ${\text{k}}$ and ${\text{z}}_{{\text{j}}}^{{\text{k}}} = 0$ otherwise. We want to find maximum likelihood estimation (MLE) of ${\uptheta }$ for which expected log-likelihood function, ${\text{E}}_{{{\text{Z}}|{\text{X}},{\uptheta }}} [\log {\text{L}}\left( {{\uptheta }:{\text{X}},{\text{Z}}} \right)]$ is optimized. After one iteration estimation parameters are updated by,

6 $${\text{a}}_{{{\text{k}},{\text{i }}}}^{{\text{T}}} = \frac{{\mathop \sum \nolimits_{{{\text{j}} \in {\text{SJ}}_{{\text{i}}}^{{\text{k}}} }} {\text{Z}}_{{\text{k}}} \left( {\text{j}} \right)}}{{\mathop \sum \nolimits_{{{\text{j}} = 1}}^{{\text{N}}} {\text{Z}}_{{\text{k}}} \left( {\text{j}} \right)}}$$

7 $${\text{d}}_{{\text{k}}} = \frac{{\mathop \sum \nolimits_{{{\text{j}} = 1}}^{{\text{N}}} {\text{Z}}_{{\text{k}}} \left( {\text{j}} \right)}}{{\text{N}}}$$

where ${\text{SJ}}_{{\text{i}}}^{{\text{k}}}$ are the time indices when ${\text{S}}_{{\text{i}}}$ measures the value to be ${\text{k}}$. ${\text{Z}}_{{\text{k}}} \left( {\text{j}} \right)$ is the probability that ${\text{C}}_{{\text{j}}}$’s value to be ${\text{k}}$ conditioned upon the input matrix ${\text{X}}$ and current estimated parameter ${\uptheta }$:

8 $${\text{Z}}_{{\text{k}}} \left( {\text{j}} \right) = {\text{P}}\left( {{\text{z}}_{{\text{j}}} = {\text{k|X}}_{{\text{j}}} ,{\uptheta }} \right) = \frac{{{\text{A}}_{{\text{k}}} \left( {\text{j}} \right) \times {\text{d}}_{{\text{k}}} }}{{\mathop \sum \nolimits_{{{\text{k}} = 1}}^{{\text{K}}} {\text{A}}_{{\text{k}}} \left( {\text{j}} \right) \times {\text{d}}_{{\text{k}}} }}$$

where

9 $${\text{A}}_{{\text{k}}} \left( {\text{j}} \right) = {\text{P}}\left( {{\text{X}}_{{\text{j}}} ,\uptheta |{\text{z}}_{{\text{j}}} = {\text{k}}} \right) = \mathop \prod \limits_{{{\text{i}} = 1}}^{{\text{M}}} ({\text{a}}_{{{\text{k}},{\text{i}}}}^{{\text{T}}} )^{{{\text{S}}_{{\text{i}}} {\text{C}}_{{\text{j}}}^{{\text{k}}} }} \times ({\text{a}}_{{{\text{k}},{\text{i}}}}^{{\text{F}}} )^{{{\text{S}}_{{\text{i}}} {\text{C}}_{{\text{j}}}^{{{\overline{\text{k}}}}} }}$$

We iteratively update ${\uptheta }$ using Eqs. (6)–(9) until the difference of ${\uptheta }$ between two consecutive iterations reaches the minimum. We define the minimum update point as the converging point. After convergence, we estimate the true value from the optimized ${\text{Z}}_{{\text{k}}} \left( {\text{j}} \right)$. Estimated value ${\text{O}}_{{\text{j}}}$ at a time index ${\text{j}}$ is the value of k for which ${\text{Z}}_{{\text{k}}} \left( {\text{j}} \right)$ is maximum. We get the credibility index ${\text{t}}_{{\text{i}}}$ of sensor ${\text{S}}_{{\text{i}}}$ for a time window by averaging ${\text{t}}_{{\text{i}}}^{{\text{k}}}$ which is calculated from optimized ${\text{a}}_{{{\text{k}},{\text{i }}}}^{{\text{T}}}$ using Eq. (4), over ${\text{k}} = 1,2, \ldots .,{\text{K}}$.

### Initialization

At each time window we start the optimization with ${\uptheta }_{{\text{k}}} = \left( {{\text{a}}_{{{\text{k}},{\text{i}}}}^{{\text{T}}} ,{\text{d}}_{{\text{k}}} } \right)$ initialized between 0 and 1. Since we do not know the true signal level a priori, ${\text{d}}_{{\text{k}}}$ is assumed to be uniformly distributed over ${\text{k}} = 1,2, \ldots .,{\text{K}}$. At the very beginning we assume all sensors are reliable. Therefore, at the first time window we initialize ${\text{a}}_{{{\text{k}},{\text{i}}}}^{{\text{T}}}$ with random values greater than 0.5 for all sensors (i.e., ${\text{a}}_{{{\text{k}},{\text{i}}}}^{{\text{T}}} = 0.5 + {\updelta }_{{\text{i}}}$ for ${\text{i}} = 1,2, \ldots ,{\text{M}}$ and ${\text{k}} = 1,2, \ldots .,{\text{K}}$, where ${\updelta }_{{\text{i}}}$ is a random number between 0 and 0.5). For the subsequent time windows, we use the previous credibility information to initialize ${\text{a}}_{{{\text{k}},{\text{i}}}}^{{\text{T}}}$ such that,

10 $${\text{a}}_{{{\text{k}},{\text{i}}}}^{{\text{T}}} = {\upbeta } \times {\text{t}}_{{{\text{i}},{\text{avg}}}} + \left( {1 - {\upbeta }} \right) \times \left( {0.5 + {\updelta }_{{\text{i}}} } \right)$$

where ${\upbeta }$ is a parameter, 0 $\le$ ${\upbeta }$ $\le$ 1 and ${\text{t}}_{{{\text{i}},{\text{avg}}}}$ is ${\text{S}}_{{\text{i}}}$ sensor’s historical credibility calculated by averaging credibility ${\text{t}}_{{\text{i}}}$ of previous time windows. The choice of ${\upbeta }$ decides how much importance we want to give to historical performance. We can set small ${\upbeta }$ when majority of the sensors in a network is reliable. However, when most of the sensors in a network is unreliable, we should rely on a few most credible sensors rather than the majority. Large ${\upbeta }$ should be chosen in such a case. We have used ${\upbeta } = 1$ for all our analyses. Only in Fig. 7a where we estimate the signal assuming equal credibility of all sensors (red circles), ${\upbeta } = 0$ was chosen.

### Theoretical credibility threshold limit (CTL)

#### CTL is 0.5 without historical credibility

We consider there are total ${\text{M}}$ sensors in a group. Number of reliable sensors is ${\text{R}}$ and unreliable sensors is ${\text{U}}$ (i.e., ${\text{R}} + {\text{U}} = {\text{M}}$). If we assume all sensors are equally reliable without considering historical credibility, we initialize estimation parameter ${\text{a}}_{{{\text{k}},{\text{i}}}}^{{\text{T}}}$, with random values greater than 0.5 (${\upbeta } = 0$ in Eq. 10). For concreteness, let us assume, ${\text{a}}_{{{\text{k}},{\text{i}}}}^{{\text{T}}} = 0.5 + {\updelta }$ for ${\text{i}} = 1,2, \ldots ,{\text{M}}$ and ${\text{k}} = 1,2, \ldots .,{\text{K}}$, where ${\updelta }$ is constant for all sensors and close to zero. We also assume ${\text{d}}_{{\text{k}}}$ is a uniformly distributed prior probability that signal value is ${\text{k}}$ at any time.

Let us take the true value of ${\text{C}}_{{\text{j}}}$ to be ${\text{k}}$. All the reliable sensors will measure ${\text{C}}_{{\text{j}}}$’s value to be ${\text{k}}$ and unreliable sensors will measure other than ${\text{k}}$. We can group together all values other than ${\text{k}}$ and call it as ${\overline{\text{k}}}$.

11 $$\begin{aligned} & {\text{S}}_{{\text{i}}} {\text{C}}_{{\text{j}}} = {\text{k}},{ }\forall {\text{ i}} \in {\text{R}} \\ & {\text{S}}_{{\text{i}}} {\text{C}}_{{\text{j}}} = {\overline{\text{k}}},{ }\forall {\text{ i}} \in {\text{U}} \\ \end{aligned}$$

Using values of Eq. (11) into Eqs. (8)–(9) and applying Taylor series expansion for ${\updelta }$ <  < 0.5,

12 $$\begin{aligned} & {\text{A}}_{{\text{k}}} \left( {\text{j}} \right) = \left( {0.5 + {\updelta }} \right)^{{\text{R}}} \times \left( {0.5 - {\updelta }} \right)^{{\text{U}}} \approx \left( {0.5} \right)^{{\text{M}}} \left( {1 + 2{\text{R}}\updelta } \right)\left( {1 - 2{\text{U}}\updelta} \right) \\ & A_{{\overline{k}}} \left( {\text{j}} \right) = \left( {0.5 - {\updelta }} \right)^{{\text{R}}} \times \left( {0.5 + {\updelta }} \right)^{{\text{U}}} \approx \left( {0.5} \right)^{{\text{M}}} \left( {1 - 2{\text{R}}\updelta} \right)\left( {1 + 2{\text{U}}\updelta } \right) \\ & \frac{{{\text{Z}}_{{\text{k}}} \left( {\text{j}} \right)}}{{{\text{Z}}_{{{\overline{\text{k}}}}} \left( {\text{j}} \right)}} = \frac{{{\text{A}}_{{\text{k}}} \left( {\text{j}} \right)}}{{{\text{A}}_{{{\overline{\text{k}}}}} \left( {\text{j}} \right)}} = \frac{{\left( {1 + 2{\text{R}}\updelta } \right)\left( {1 - 2{\text{U}}\updelta } \right)}}{{\left( {1 - 2{\text{R}}\updelta } \right)\left( {1 + 2{\text{U}}\updelta } \right)}} = \frac{{\frac{1}{{\text{U}}} + 2\frac{{\text{R}}}{{\text{U}}}{\updelta }}}{{\frac{1}{{\text{U}}} + 2{\updelta }}} \times \frac{{\frac{1}{{\text{U}}} - 2{\updelta }}}{{\frac{1}{{\text{U}}} - 2\frac{{\text{R}}}{{\text{U}}}{\updelta }}} \\ \end{aligned}$$

Recall that ${\text{R}} + {\text{U}} = {\text{M}}$, is the total number of sensors. If number of reliable sensors is larger than unreliable sensors ($\frac{{\text{R}}}{{\text{U}}} > 1$ i.e., $\frac{{\text{U}}}{{\text{M}}} < 0.5$), from Eq. (12), ${\text{Z}}_{{\text{k}}} \left( {\text{j}} \right) > {\text{Z}}_{{{\overline{\text{k}}}}} \left( {\text{j}} \right)$. Since estimated value is the one for which ${\text{Z}}_{{\text{k}}} \left( {\text{j}} \right)$ is the maximum, estimated value is the actual value ${\text{k}}$. Similarly, calculated credibility ${\text{t}}_{{\text{i}}}$ will be larger for reliable sensors than unreliable ones according to Eq. (4) and 6. Therefore reliable sensors will be assigned higher credibility than unreliable sensors. However, if number of reliable sensors is smaller than unreliable sensors ($\frac{{\text{R}}}{{\text{U}}} < 1$ i.e., $\frac{{\text{U}}}{{\text{M}}} > 0.5$), then ${\text{Z}}_{{\text{k}}} \left( {\text{j}} \right) < {\text{Z}}_{{{\overline{\text{k}}}}} \left( {\text{j}} \right)$. Therefore, estimated signal will be ${\overline{\text{k}}}$ whereas actual value is ${\text{k}}$. Also calculated credibility ${\text{t}}_{{\text{i}}}$ for reliable sensors will be smaller than unreliable sensors in this case.

This analysis shows theoretical CTL of MLE is 0.5 without historical credibility. That means, at least half of the total sensors must be reliable to estimate the true signal which is equivalent to majority voting (${\upalpha } = 0.5$ in Eq. 2). We also see from Fig. 7a that MLE fails to estimate the true signal and RMSE increases rapidly at $\frac{{\text{U}}}{{\text{M}}} = 0.5$ when historical credibility is not used (red circles).

#### CTL exceeds 0.5 with historical credibility

When we use historical credibility information for initialization of ${\text{a}}_{{{\text{k}},{\text{i}}}}^{{\text{T}}}$ (${\upbeta } = 1$ in Eq. 10), we give more weight to reliable sensors. For theoretical calculation, we assume the initialization of ${\text{a}}_{{{\text{k}},{\text{i}}}}^{{\text{T}}}$ is—${\text{a}}_{{{\text{k}},{\text{i}}}}^{{\text{T}}} = 0.5 + {\updelta },{ }\forall {\text{ i}} \in {\text{R}}$ (reliable sensors), and ${\text{a}}_{{{\text{k}},{\text{i}}}}^{{\text{T}}} = 0.5 - {\updelta },{ }\forall {\text{ i}} \in {\text{U}}$ (unreliable sensors). For the same problem mentioned above, we get from Eqs. (8)–(9),

13 $$\begin{aligned} & {\text{A}}_{{\text{k}}} \left( {\text{j}} \right) = \left( {0.5 + {\updelta }} \right)^{{\text{R}}} \times \left( {0.5 + {\updelta }} \right)^{{\text{U}}} \approx \left( {0.5} \right)^{{\text{M}}} \left( {1 + 2{\text{R}}\updelta} \right)\left( {1 + 2{\text{U}}\updelta} \right) \\ & {\text{A}}_{{{\overline{\text{k}}}}} \left( {\text{j}} \right) = \left( {0.5 - {\updelta }} \right)^{{\text{R}}} \times \left( {0.5 - {\updelta }} \right)^{{\text{U}}} \approx \left( {0.5} \right)^{{\text{M}}} \left( {1 - 2{\text{R}}\updelta } \right)\left( {1 - 2{\text{U}}\updelta } \right) \\ & \frac{{{\text{Z}}_{{\text{k}}} \left( {\text{j}} \right)}}{{{\text{Z}}_{{{\overline{\text{k}}}}} \left( {\text{j}} \right)}} = \frac{{{\text{A}}_{{\text{k}}} \left( {\text{j}} \right)}}{{{\text{A}}_{{{\overline{\text{k}}}}} \left( {\text{j}} \right)}} = \frac{{\left( {1 + 2{\text{R}}\updelta } \right)\left( {1 + 2{\text{U}}\updelta } \right)}}{{\left( {1 - 2{\text{R}}\updelta } \right)\left( {1 + 2{\text{U}}\updelta } \right)}} = \frac{{\frac{1}{{\text{U}}} + 2\frac{{\text{R}}}{{\text{U}}}{\updelta }}}{{\frac{1}{{\text{U}}} - 2{\updelta }}} \times \frac{{\frac{1}{{\text{U}}} + 2{\updelta }}}{{\frac{1}{{\text{U}}} - 2\frac{{\text{R}}}{{\text{U}}}{\updelta }}} \\ \end{aligned}$$

From Eq. (13), we can see ${\text{Z}}_{{\text{k}}} \left( {\text{j}} \right) > {\text{Z}}_{{{\overline{\text{k}}}}} \left( {\text{j}} \right)$ for both $\frac{{\text{R}}}{{\text{U}}} > 1$($\frac{{\text{U}}}{{\text{M}}} < 0.5$) and $\frac{{\text{R}}}{{\text{U}}} < 1$($\frac{{\text{U}}}{{\text{M}}} > 0.5$) conditions. Therefore, estimated value is true value k even if number of reliable sensors is less than unreliable sensors. This implies using historical credibility, CTL of MLE can achieve theoretical upper limit (${\upalpha } = 1$ in Eq. 2). Indeed, we numerically obtain CTL of a real sensor dataset greater than 0.8 using historical credibility (shown in Fig. 7c). In the theoretical analysis, we assume all the unreliable sensors agree at a single level (i.e., ${\overline{\text{k}}}$) which is a conservative assumption. In practice all unreliable sensors may not agree at a single level which will also help to achieve higher CTL.

## Supplementary Information

Supplementary Information.

## Data Availability

All data are provided either in main manuscript or in supplementary information.
