# Supplementary material for: A new paradigm of reliable sensing with field-deployed electrochemical sensors integrating data redundancy and source credibility
Source: Sci Rep. 2023 Feb 22;13:3101. doi: 10.1038/s41598-022-25920-w (PMC9946936; doi:10.1038/s41598-022-25920-w)
Supplement: Supplementary file 1 — Supplementary Information. [file 41598_2022_25920_MOESM1_ESM.pdf]

Supplementary Information

A New Paradigm of Reliable Sensing with Field-deployed Electrochemical Sensors Integrating Data Redundancy and Source Credibility

Ajanta Saha[1], Sotoudeh Sedaghat[2,3], Sarath Gopalakrishnan[1], Jose Waimin[2], Aiganym Yermembetova[2], Nicholas Glassmaker[3], Charilaos Mousoulis[1], Ali Shakouri[1], Alexander Wei[2,3,4], Rahim Rahimi[2], and Muhammad A. Alam[1*]

[1] School of Electrical and Computer Engineering, Purdue University, West Lafayette, IN 47906,USA
[2] Department of Materials Engineering, Purdue University, West Lafayette, IN 47907, USA
[3] Birck Nanotechnology Center, Purdue University, West Lafayette, IN 47907, USA
[4] Department of Chemistry, Purdue University, West Lafayette, IN 47906, USA
* Corresponding Author: Muhammad A. Alam (alam@purdue.edu)

Supplementary Information Text

S1. Synthetic data generation: Synthetic data are generated by analytical model of solid contact Ion selective electrode (ISE) and faults are injected manually into output of unreliable sensors.

A. Ideal response of Nernst based ISE: Potentiometric ISE sensor generates potential as a function of analyte concentration based on Nernst principle (1). If target ion concentration is n, transient potential is induced following Eq. S1, followed by a steady-state response given by Eq. S2 (2),

$$V_{transient}(t) = \frac{k_B T}{zq} \ln\left[\frac{t(n - n_0)}{\frac{C_1 \kappa \epsilon_0}{zqD}\frac{k_B T}{zq} + tn} + 1\right] + V_0 \qquad (S1)$$

$$V_{steady-state} = \frac{k_B T}{zq} \ln\left[\frac{nh^2}{C_2 \kappa}\right] + C_3 \qquad (S2)$$

where, k_B is the Boltzmann's constant, q is elementary charge, T is the solution temperature, z is target ion charge number, $\kappa\epsilon_0$ is dielectric constant of the sensing layer, and C_1, C_2, C_3 are constants. V_0 is the initial potential corresponding to initial concentration n_0. D, h are associated with physical and chemical properties of ISE where, D is diffusion coefficient of target ion in the ISE, h is thickness of the sensing layer. We calculate ideal response of ISEs from Eq. S1, S2 by varying target ion's concentration n from 10^{-4} M to 10^{-1} M in discrete steps.

B. Fault injection: Eq. S1, S2 shows transient and steady-state potential of ISE depend on its physical and chemical properties (i.e., D, h). Therefore, in the field application if the sensor properties change due to degradation, the corresponding potential will change. To simulate the degradation, we keep D and h constant for reliable sensors whereas change D or h for unreliable sensors. We reduce the diffusion coefficient of sensor 8 to 10 ($S_8 - S_{10}$) from initial D = 10^{-13} m^2/s to 10^{-16} m^2/s in the time period 300 s-480 s which causes delayed response of the sensors during that time. We also reduce h of sensor 1 to 3 ($S_1 - S_3$) from h =20 um to 12 um in the time period 480 s-740 s which causes a constant shift of output voltage. Thickness of sensor 4 (S_4) is made 0 at t=640s to simulate the effect of delamination which makes sensors insensitive to concentration change. Moreover, we simulate the effect of extrinsic factors by injecting random faults such as

drift and malfunction error to $S_8 - S_{10}$ in the time period 0-160 s using standard algorithm (3, 4). Output voltage of reliable and unreliable sensors of synthetic dataset is shown in Fig.2.

S2. Pseudocode of true signal and credibility estimation:

Algorithm 1

function *signal-credibility-estimation* (A, $\mathbf{t_{i,avg}}$)

Input: A is a matrix containing continuous time signal of $\mathbf{M}$ sensors; $\mathbf{t_{i,avg}}$ is their historical credibility

Output: Estimated signal, average credibility

# define parameters

$\mathbf{N}$ ← window size

$\mathbf{t_{ovl}}$ ← overlap period

$\mathbf{N_{ovl}}$ ← number of overlapping windows

$\mathbf{K}$ ← quantization level

$\mathbf{R_{th}}$ ← reliable threshold

for n **in** $\mathbf{N_{ovl}}$ **do**

$\mathbf{SC}$ ← $\mathbf{M}$ sensors signal for $\mathbf{N}$ time steps at n^th window

# Quantization

$\mathbf{k}$ ← divide min ($\mathbf{SC}$) and max ($\mathbf{SC}$) in $\mathbf{K}$ levels

$\mathbf{SC}$ ← $\mathbf{SC}$ converted to nearest $\mathbf{k}$ value

# Initialization

$\mathbf{d_k = uniform\{1, K\}}$

for $\mathbf{i}$ **in** $\mathbf{M}$ **do**

if $\mathbf{t_{i,avg}}$ **is** present **then**

$\mathbf{a_{k,i}^T = t_{i,avg}}$

else

$\mathbf{a_{k,i}^T = 0.5 + random(0, 0.5)}$

end if

end for

# True signal and credibility estimation

Estimated signal, credibility at n^th window ← ***MLE*** ($\mathbf{SC}$, $\mathbf{k}$, $\mathbf{d_k}$, $\mathbf{a_{k,i}^T}$) **# for details see Methods**

Estimated signal ← concatenate Estimated signal of nth window

$\mathbf{t_{i,avg}}$ = mean (credibility at n^th window, $\mathbf{t_{i,avg}}$)

# drift correction

for $\mathbf{i}$ **in** $\mathbf{M}$ **do**

if $\mathbf{t_{i,avg} < R_{th}}$ **then**

error signal ← Estimated signal – sensor signal of nth window

sensor signal ←sensor signal + mean (error signal)

end if

end for

end for

return Estimated signal, $\mathbf{t_{i,avg}}$

S3. Field deployment of nitrate ISEs:

For field deployment, ISEs are packaged by placing them within 3D printed containers that is filled with weather-proof epoxy compound as encapsulant. Packaged sensors are interfaced with LoRa (Long Range low-power, wide-area) boards for wireless communication as shown in Figure. 5. Prior to field deployment, ISEs were conditioned in a solution of 0.1 M KNO_3 until their voltage output is stabilized and Nernst response is achieved. For data collection, packaged sensors were deployed at the Agronomy Center for Research and Education (ACRE) of Purdue University which has a water quality field station to collect water washed away from agricultural field. Sensors were placed in 600 mL containers inside the water-quality field stations. Water from adjacent fields was collected automatically in a large container and then transferred into the containers where ISEs were placed. Nitrate sensors monitored the nitrate concentration in the sampled water continuously for 22 days. To obtain the ground truth measurements of nitrate concentration periodically, automated colorimetry based on EPA (US Environmental Protection Agency) Method 353.2 was used (5).

S4. Pseudocode of data transmission approach based on credibility:

Algorithm 2

```
Input: Continuous time signal of M sensors
Output: Transmitted datapoints
# define event detection threshold
y ← event detection threshold
# initialize credibility of the sensors
Estimated signal, credibility ← signal-credibility-estimation (M sensors signal for a time period)
top credible sensor← sensor with the highest credibility
for each time step do
    # event monitoring by top credible sensor
    if current datapoint of top credible sensor differs by > y% of last transmitted datapoint then
        Detect current datapoint as event
    end if
    if event is detected then
        Collect all sensors signal for a time window
        # check and update credibility of the sensors
        Estimated signal, credibility ← signal-credibility-estimation (M sensors signal for
                                                    the time period, credibility)
        top credible sensor← sensor with the highest credibility
        if sensor that detected event is top credible then
            Transmit Estimated signal
        end if
    end if
end for
```

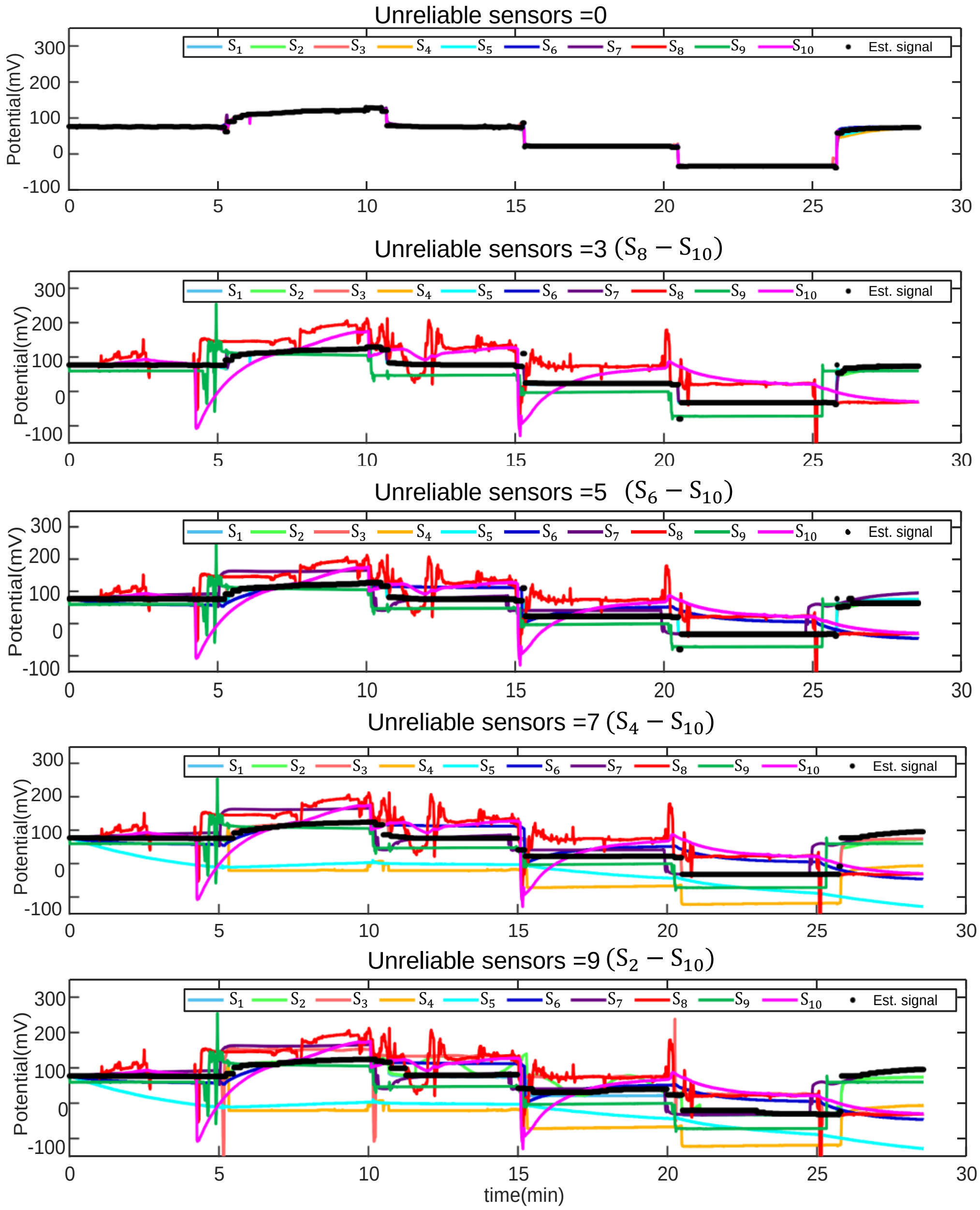

Fig. S1. Recorded voltage of nitrate sensors in controlled experiment. Some sensors show unreliable voltage reading due to random faults and gradual drift. We chose different number of unreliable sensors (0-9) in the dataset of total 10 sensors. Number of unreliable sensors and the corresponding sensor ids are indicated for each dataset. Estimated signal is calculated by MLE approach using historical credibility information ($\beta = 1$). The dataset is used for analyses of Fig.6-7.

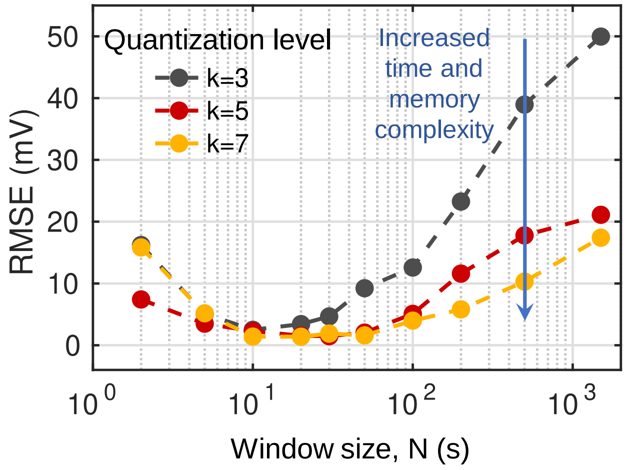

Fig. S2. RMSE between estimated signal (with drift correction) of the sensors shown in Fig. 4(a) and true signal as a function of window size, N and quantization level, K.

Table S1. Event detection thresholds used for data transmission analysis of real sensor datasets.

Real sensor datasets	Event detection threshold (y%)
Gradual drift	8
Random faults	4
Field	5

Table S2. Window size, overlap period and quantization level used for synthetic and real sensor datasets.

datasets	Widow size (N)	Overlap period	Quantization level (K)
Synthetic	10 s	2 s	3
Gradual drift	25 s	5 s	3
Random faults	20 s	5 s	3
Field	9 hour	5 hour	3

SI References

1. Bard Allen J., Faulkner Larry R., "Potentials and Thermodynamics of Cells" in *Electrochemical Methods Fundamentals and Applications*, pp. 44–85.

2. X. Jin, *et al.*, Steady-State and Transient Performance of Ion-Sensitive Electrodes Suitable for Wearable and Implantable Electro-Chemical Sensing. *IEEE Transactions on Biomedical Engineering* **69**, 96–107 (2022).

3. K. Ni, *et al.*, Sensor Network Data Fault Types. *ACM Transactions on Sensor Networks* **5**, 1–29 (2009).

4. B. de Bruijn, T. A. Nguyen, D. Bucur, K. Tei, Benchmark datasets for fault detection and classification in sensor data in *SENSORNETS 2016 - Proceedings of the 5th International Confererence on Sensor Networks*, (SciTePress, 2016), pp. 185–195.

5. W. O. James, “Method 353.2, Revision 2.0: Determination of Nitrate-Nitrite Nitrogen by Automated Colorimetry” (1993).
